# Enhancer of Zeste Homologue 2 Inhibition Attenuates TGF-β Dependent Hepatic Stellate Cell Activation and Liver Fibrosis

**DOI:** 10.1016/j.jcmgh.2018.09.005

**Published:** 2018-09-15

**Authors:** Rosa Martin-Mateos, Thiago M. De Assuncao, Juan Pablo Arab, Nidhi Jalan-Sakrikar, Usman Yaqoob, Thomas Greuter, Vikas K. Verma, Angela J. Mathison, Sheng Cao, Gwen Lomberk, Philippe Mathurin, Raul Urrutia, Robert C. Huebert, Vijay H. Shah

**Affiliations:** 1 Division of Gastroenterology and Hepatology, Mayo Clinic, Rochester, Minnesota; 2 Division of Gastroenterology and Hepatology, Ramón y Cajal University Hospital, Madrid, Spain; 3 Departamento de Gastroenterologia, Escuela de Medicina, Pontificia Universidad Catolica de Chile, Santiago, Chile; 4 Genomics and Precision Medicine Center (GSPMC), Medical College of Wisconsin, Milwaukee, Wisconsin; 5 Division of Research, Department of Surgery, Medical College of Wisconsin, Milwaukee, Wisconsin; 6 Service Maladie de l'Appareil Digestif, INSERM U995 Université Lille 2, Centre Hospitalier Régionale Universitaire (CHRU) de Lille, France

**Keywords:** EZH2, Liver Fibrosis, Epigenetics, Histone Modifications, BDL, bile duct ligation, CCL_4_, carbon tetrachloride, ChIP, chromatin immunoprecipitation, CTGF, connective tissue growth factor, DKK1, Dickkopf-1, ECM, extracellular matrix, EZH2, enhancer of zeste homologue 2, FBS, fetal bovine serum, FDR, false discovery rate, H3K27me3, trimethylation of histone 3 at lysine 27, HCC, hepatocellular carcinoma, HMT, histone methyltransferase, HSC, hepatic stellate cell, IP, intraperitoneally, IPA, Ingenuity Pathway Analysis, logFC, logarithmic fold change, mRNA, messenger RNA, PCR, polymerase chain reaction, PDGF, platelet-derived growth factor, siRNA, small interfering RNA, α-SMA, α-smooth muscle actin, TGF-β, transforming growth factor β, VEGFA, vascular endothelial growth factor A, WT, wild type

## Abstract

**Background & Aims:**

Transdifferentiation of hepatic stellate cells (HSCs) into myofibroblasts is a key event in the pathogenesis of liver fibrosis. Transforming growth factor β (TGF-β) and platelet-derived growth factor (PDGF) are canonical HSC activators after liver injury. The aim of this study was to analyze the epigenetic modulators that differentially control TGF-β and PDGF signaling pathways.

**Methods:**

We performed a transcriptomic comparison of HSCs treated with TGF-β or PDGF-BB using RNA sequencing. Among the targets that distinguish these 2 pathways, we focused on the histone methyltransferase class of epigenetic modulators.

**Results:**

Enhancer of zeste homolog 2 (EZH2) was expressed differentially, showing significant up-regulation in HSCs activated with TGF-β but not with PDGF-BB. Indeed, EZH2 inhibition using either a pharmacologic (GSK-503) or a genetic (small interfering RNA) approach caused a significant attenuation of TGF-β–induced fibronectin, collagen 1α1, and α-smooth muscle actin, both at messenger RNA and protein levels. Conversely, adenoviral overexpression of EZH2 in HSCs resulted in a significant stimulation of fibronectin protein and messenger RNA levels in TGF-β–treated cells. Finally, we conducted in vivo experiments with mice chronically treated with carbon tetrachloride or bile duct ligation. Administration of GSK-503 to mice receiving either carbon tetrachloride or bile duct ligation led to attenuated fibrosis as assessed by Trichrome and Sirius red stains, hydroxyproline, and α-smooth muscle actin/collagen protein assays.

**Conclusions:**

TGF-β and PDGF share redundant and distinct transcriptomic targets, with the former predominating in HSC activation. The EZH2 histone methyltransferase is preferentially involved in the TGF-β as opposed to the PDGF signaling pathway. Inhibition of EZH2 attenuates fibrogenic gene transcription in TGF-β–treated HSCs and reduces liver fibrosis in vivo. The data discussed in this publication have been deposited in NCBI's Gene Expression Omnibus and are accessible through GEO Series accession number GSE119606 (https://www.ncbi.nlm.nih.gov/geo/query/acc.cgi?acc=GSE119606)

## Graphical abstract

**Figure:**
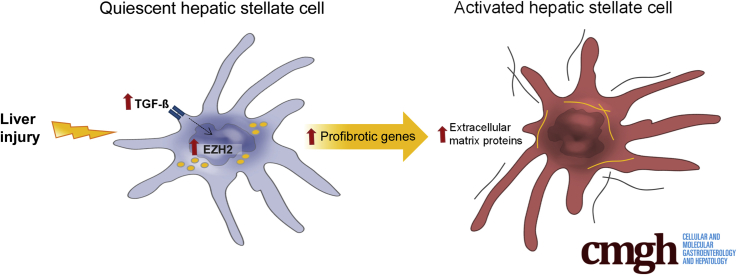

See editorial on page 237.

**Summary**

The histone methyltransferase enhancer of zeste homologue 2 promotes transforming growth factor–β–dependent hepatic stellate cell activation. Consistent with this function, enhancer of zeste homologue 2 inhibition attenuates stellate cell activation and fibrosis induced by carbon tetrachloride or bile duct ligation.

Cirrhosis, as the last stage of chronic liver diseases, is characterized by the accumulation of extracellular matrix (ECM), chronic inflammation, and fibrosis.^1^ Hepatic stellate cells (HSCs) constitute the primary source of ECM once they transdifferentiate into myofibroblasts.^2^ Transforming growth factor β (TGF-β) and platelet-derived growth factor (PDGF) are key growth factor ligands that drive the transdifferentiation process.^3^ TGF-β binding to the type I receptor induces phosphorylation of downstream SMAD proteins, which ultimately promotes transcription of matrix components.^4^ PDGF, on the other hand, is one of the best-known mitogens for HSCs.^5^ A better understanding of the redundant and distinct pathways that control TGF-β and PDGF-dependent HSC activation may lead to more refined approaches for potential therapeutic benefit.

Epigenetics defines the reversible and inheritable changes in gene expression that do not alter the underlying DNA sequence.^6^ Epigenetic modifications act coordinately to configure cell type and context-specific gene transcription programs. DNA methylation, noncoding RNAs, and histone modifications encompass mechanisms of epigenetic regulations. With regard to the latter, histone modifications such as methylation, acetylation, ubiquitination, or phosphorylation lead to reversible changes in chromatin structure and consequently to transcriptional gene activation or repression.^7^ Enhancer of zeste homologue 2 (EZH2) is a histone methyltransferase responsible for the trimethylation of histone 3 at lysine 27 (H3K27me3). This epigenetic mark promotes chromatin compaction and silencing of gene transcription.^8^ EZH2 mediates transcriptional repression of several tumor-suppressor genes,^9^, ^10^ promoting proliferation and metastasis. Some evidence also suggests that EZH2 may promote fibrosis,^11^, ^12^, ^13^ but its specific role in the TGF-β signaling pathway during HSC transdifferentiation has not been addressed.

In this study, we first sought to characterize the epigenetic mechanisms involved in TGF-β– vs PDGF-specific signaling pathways during HSC activation. For an unbiased approach, we first performed RNA sequencing to compare gene expression profiles in primary human HSCs treated with either TGF-β or PDGF. We found that among histone methyltransferases (HMTs), EZH2 expression increased in HSCs treated with TGF-β but not with PDGF, which led us to hypothesize that EZH2 is specifically involved in TGF-β dependent profibrotic pathways. To further explore the role of EZH2 in fibrogenesis, we tested the effect of either genetic (small interfering RNA [siRNA]) or pharmacologic (GSK-503) EZH2 inhibition. Our results show that EZH2 inhibition attenuates fibrogenic gene transcription and protein expression in TGF-β–activated HSCs. Conversely, EZH2 adenoviral overexpression promoted production of matrix proteins. In vivo, we found that EZH2 inhibition attenuates liver fibrosis in the carbon tetrachloride (CCL_4_) and bile duct ligation (BDL) murine models. This work provides further understanding of the complex network of epigenetic changes that occur during liver fibrogenesis and shows the profibrotic role of EZH2 in HSCs in vitro and in vivo. Our findings suggest that targeting epigenetic modulators involved in HSC activation such as EZH2 could be useful for future liver fibrosis therapies.

## Results

### TGF-β Shares Overlapping Targets With PDGF but Has a More Dominant Role in HSC Activation

**Figure 1:**
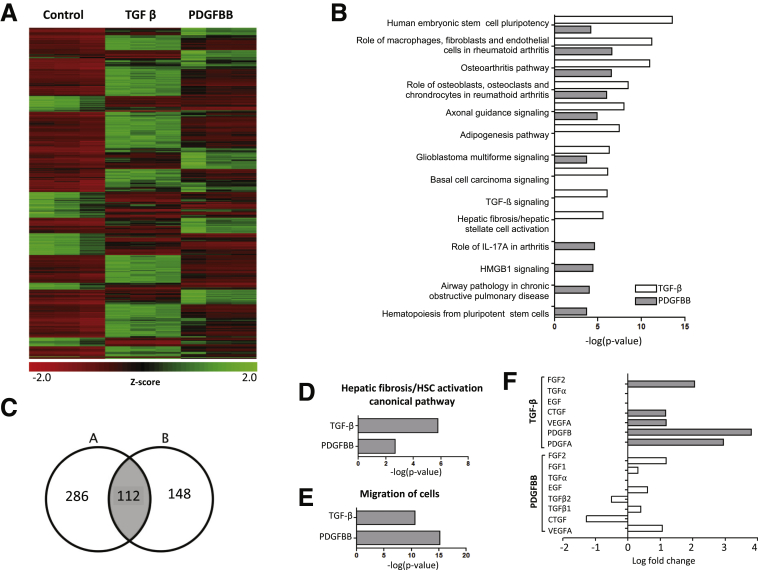
**TGF-β shares overlapping targets with PDGF but has a predominant role in early HSC activation.** Primary human HSCs were treated with either TGF-β or PDGF-BB for 2 hours. RNA sequencing was performed. (*A*) Whole-genome expression heat map presenting basal gene expression and changes after TGF-β and PDGF-BB stimulation showed overlapping regulation of multiple genes. (*B*) IPA showed overlapping roles regarding the top 10 canonical pathways activated after TGF-β and PDGF-BB treatment. (*C*) By using FDR of 0.05 and logFC >1.5 as selection criteria, a Venn diagram showed that TGF-β and PDGF concurred in the regulation of 112 different genes implicated in a wide range of biological functions. (*D*) Hepatic fibrosis and the HSC activation pathway was more relevant for the TGF-β data set (5.8 –log[*P* value]). (*E*) Cell migration activity was increased significantly either with TGF-β (-log[*P* value], 10.670) or PDGF-BB (15.185 -log[*P* value]). (*F*) Comparison analysis of different growth factors showed that TGF-β stimulation induces up-regulation of PDGFA (logFC, 2.949), PDGFB (logFC, 3.806), VGFA (logFC, 1.1836), CTGF (logFC, 1.163), and fibroblast growth factor 2 (FGF2) (logFC, 2.0640). Results from 3 independent experiments each performed in triplicate are shown. EGF, epidermal growth factor; IL, interleukin.

To compare TGF-β and PDGF signaling pathways using an unbiased approach, we performed RNA sequencing in primary human HSCs treated with TGF-β or PDGF-BB for 2 hours after overnight serum starvation. A whole-genome expression heat map comparing basal vs TGF-β and PDGF-BB stimulation gene expression showed that these dominant HSC regulatory growth factors overlap in terms of the activation and repression of multiple targets (Figure 1*A*). The analysis of the top 10 activated canonical pathways using Ingenuity Pathway Analysis (IPA, Qiagen Inc, Hilden, Germany) software also showed substantial overlapping roles (Figure 1*B*). A Venn diagram confirmed that both concur on the significant regulation of 112 specific genes (Table 1) implicated in a wide range of biological activities (gene selection criteria: log fold change >1.5 and false discovery rate [FDR] < 0.05) (Figure 1*C*). However, the hepatic fibrosis/HSC activation pathway was highly significant for the TGF-β data set (-log[*P* value], 5.6), whereas treatment with PDGF-BB was not as dominant as TGF-β for this domain (-log[*P* value], 2.686) (Figure 1*D*). Interestingly, HSC stimulation with both TGF-β and PDGF-BB showed increased cell migration activity (TGF-β -log[*P* value], 10.670; and PDGF-BB -log[*P* value], 15.185) (Figure 1*E*). In addition, TGF-β treatment led to a significant increase in other fibrogenic growth factors such as PDGFA (2.949 logarithmic fold change [logFC]), PDGFB (3.806 logFC), vascular endothelial growth factor (VEGFA) (1.1836 logFC), connective tissue growth factor (CTGF) (1.163 logFC), or fibroblast growth factor 2 (2.0640 logFC). On the other hand, PDGF had little effect on TGF-β (TGF-β1, 0.398 logFC; TGF-β2, -0.503 logFC) or VEGFA expression (1.061 logFC), and also led to a decrease in CTGF (-1.271 logFC) (Figure 1*F*). All of these observations support the key role of TGF-β in HSC activation, which may predominate over PDGF effects despite sharing multiple target genes based on RNA sequencing.

**Table 1:** Alphabetical List of Genes Regulated by Both TGF-β and PDGF-BB

*ADM*	*DKK1*	*HES4*	*NFATC2*	*RAP1GAP2*
*AMOTL2*	*E2F7*	*HEYL*	*NIPAL4*	*RASD1*
*ANGPTL4*	*EFR3B*	*HOXB-AS2*	*NKX3-1*	*RHBDL3*
*ANKRD33B*	*EGR3*	*IFIT2*	*NPTX1*	*RHOB*
*ARAP2*	*ELFN1*	*IFNE*	*NR4A1*	*RPL39P5*
*AXIN2*	*ENC1*	*IL6*	*NR4A3*	*RRAD*
*BBC3*	*EPHB3*	*IL11*	*OLFM2*	*RUNX1T1*
*BMF*	*ESM1*	*INHBA*	*PCDH1*	*SCG2*
*BMP2*	*FAM131B*	*KCNG1*	*PDGFA*	*SEMA7A*
*BMP4*	*FAM196A*	*KCNN4*	*PDGFB*	*SPDL1*
*C3orf52*	*FAM196B*	*KIAA1644*	*PGBD5*	*SPHK1*
*C8orf4*	*FBXO32*	*KIT*	*PITPNM3*	*SPRY2*
*CACNA1G*	*FGF18*	*KREMEN2*	*PKP1*	*STC1*
*CARMIL2*	*FGFR3*	*LIF*	*PLAUR*	*TAGLN3*
*CCNG2*	*FOSB*	*LOC401472*	*PLCH2*	*TCF7*
*CEBPD*	*GAL*	*LOC541472*	*PLEKHF1*	*TFPI2*
*CECR6*	*GAS1*	*LOC105376292*	*PNP*	*TNFAIP8L3*
*CLDN4*	*GCSAM*	*LRRC8C*	*PNRC1*	*TRAF1*
*CNKSR3*	*GEM*	*LURAP1L*	*PODXL*	*WNT7B*
*CREBRF*	*GFPT2*	*MGC20647*	*PTCH1*	*ZNF365*
*CTTNBP2*	*GPR3*	*MIR17HG*	*PTGS2*	
*CXCL8*	*GREM2*	*MMP1*	*PTHLH*	
*DDIT4*	*HBEGF*	*NFATC1*	*PTPRE*	

NOTE. LogFC > 1.5 and FDR < 0.05.

FGF, fibroblast growth factor.

### EZH2 Is Up-Regulated in HSCs Treated With TGF-β but Not With PDGF-BB

**Figure 2:**
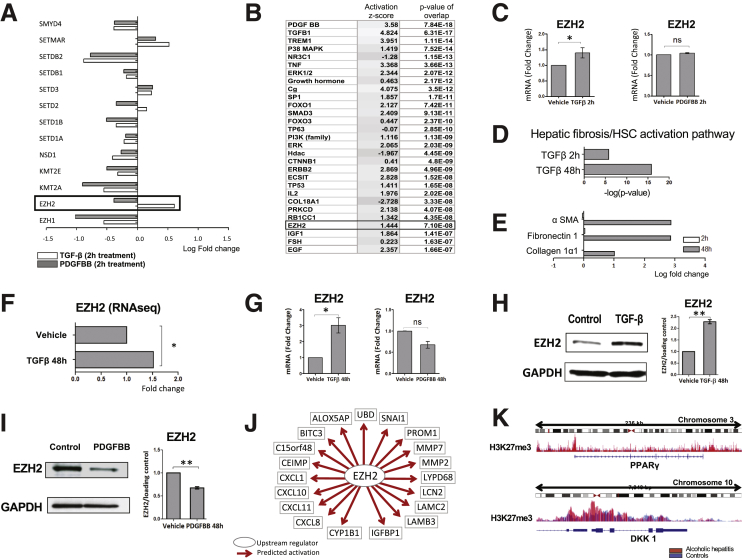
**EZH2 is up-regulated in HSCs treated with TGF-β but not with PDGF-BB.** (*A*) Differential expression analysis of HMTs in the RNA sequencing showed that EZH2 is up-regulated significantly after TGF-β but not PDGF-BB treatment (only HMTs with *P* < .05 and FDR < 0.05 are shown). (*B*) IPA identified EZH2 as an upstream regulator in HSCs treated with TGF-β (activation z-score, 1.444) (*C*) Quantitative PCR analysis confirmed that EZH2 is up-regulated after stimulation with TGF-β (∗*P* = .0286), although there were no differences with PDGF-BB (NS: *P* = .1) at 2 hours. (*D*) RNA sequencing of HSCs treated with TGF-β for 48 hours confirmed an increasing profibrotic effect by means of higher activation of the hepatic fibrosis canonical pathway and (*E*) increased significant expression of profibrotic genes: α-SMA (logFC, 2.785; *P* = 1.25E-161), fibronectin 1 (logFC, 2.860; *P* = 5.95E-111), and collagen 1α1 (logFC, 1.002; *P* = 2,04E-19). (*F*) RNA sequencing of HSCs treated with TGF-β for 48 hours confirmed the consistent up-regulation of EZH2 (*P = 4.50E-14 and FDR = 3,76E-13). (*G*) RNA sequencing results were confirmed by quantitative PCR (NS: *P* > .05; *P < .05). (*H* and *I*) Western blots and quantification graphs were consistent with previous results showing higher EZH2 expression after treatment with TGF-β (**P = .0048) vs PDGF-BB (**P = .006) at 48 hours. (*J*) RNA sequencing analysis performed in human liver samples with alcoholic hepatitis (n = 7) and controls (n = 5). IPA identified EZH2 as an important upstream regulator in alcoholic patients (activation z-score, 2.453; *P* value of overlap = 3.92E-10). Up-regulated genes consistent with activation of EZH2 are shown. (*K*) ChIP sequencing analysis confirmed a gain in H3K27me3 at the promoter of peroxisome proliferator-activated receptor γ (PPARγ) (*top panel*) and DKK1 (*bottom panel*) in this cohort (shown in red) as compared with controls (blue). The y-axis represents peak values of the histogram, and the x-axis shows the chromosomal location of the gene. Results from 3 independent experiments each performed in triplicate are shown. Paired *t* test. Data are expressed as means ± SEM. GAPDH, glyceraldehyde-3-phosphate dehydrogenase.

Given our interest in epigenetic mediators and, specifically, histone modifications, we examined differences in this domain. We found that, in particular, HMT EZH2 was expressed differentially after TGF-β and PDGF-BB treatment, with a significant increase in expression after treatment with TGF-β (fold change, 1.52; *P* = 8.25E-11; FDR, 6.95E-10), but not with PDGF (fold change, -0.77, *P* = 2.76E-04; FDR, 9.90E-4) (Figure 2*A*). By using the following as selection criteria: log fold change, ≥1.5; *P* < .05, and FDR < 0.05, IPA identified EZH2 as an upstream regulator (activation z-score, 1.444) (Figure 2*B*), which supports that EZH2 is a mediator in TGF-β–driven HSC activation. These findings were replicated at the messenger RNA (mRNA) level (Figure 2*C*). Next, to evaluate temporal kinetics of TGF-β induction of EZH2 and its other transcriptional targets, we repeated RNA sequencing of primary human HSCs treated with TGF-β for 48 hours. Comparison analysis between the TGF-β effect at 2 vs 48 hours in HSCs showed greater activation of the hepatic fibrosis/HSC activation pathway (Figure 2*D*), which correlated with up-regulation of key myofibroblastic and fibrogenic genes such as α-smooth muscle actin (α-SMA) (logFC, 2.785; *P* = 1.25E-161), fibronectin 1 (logFC, 2.860; *P* = 5.95E-111), or collagen 1α1 (logFC, 1.002; *P* = 2,04E-19) at 48 hours (Figure 2*E*). The results also showed a sustained up-regulation of EZH2 at this time point as well (fold change, 1.6; *P* = 3.89E-09; FDR, 7.86E-08) (Figure 2*F*). The changes in EZH2 expression with TGF-β and PDGF at 48 hours were confirmed at the mRNA and protein levels (Figure 2*G–I*). Finally, to translate these findings into a clinical context, we analyzed liver tissue from healthy controls (n = 5) and explants from patients with alcoholic hepatitis undergoing early liver transplantation (n = 7).^14^ mRNA sequencing and chromatin immunoprecipitation (ChIP) sequencing for H3K27me3 was performed and the epigenetic and transcriptional profiles were analyzed. The RNA sequencing analysis using IPA identified EZH2 as a highly significant upstream regulator in patients with alcoholic hepatitis (activation z-score, 2.453; *P* value of overlap = 3.92 E-10). Up-regulated genes consistent with activation of EZH2 are shown in Figure 2*J*. ChIP sequencing confirmed a gain in H3K27me3 at the promoter region of peroxisome proliferator-activated receptor γ and Dickkopf-1 (DKK1) in patients with alcoholic hepatitis as compared with healthy controls. These genes have been described previously as negative regulators of liver fibrosis (Figure 2*K*). Together, these data highlight important distinctions of TGF-β and PDGF RNA epigenetic targets including TGF-β selectivity for induction of EZH2 expression and its role in liver fibrosis.

### EZH2 Inhibition Attenuates TGF-β–Dependent HSC Activation In Vitro

**Figure 3:**
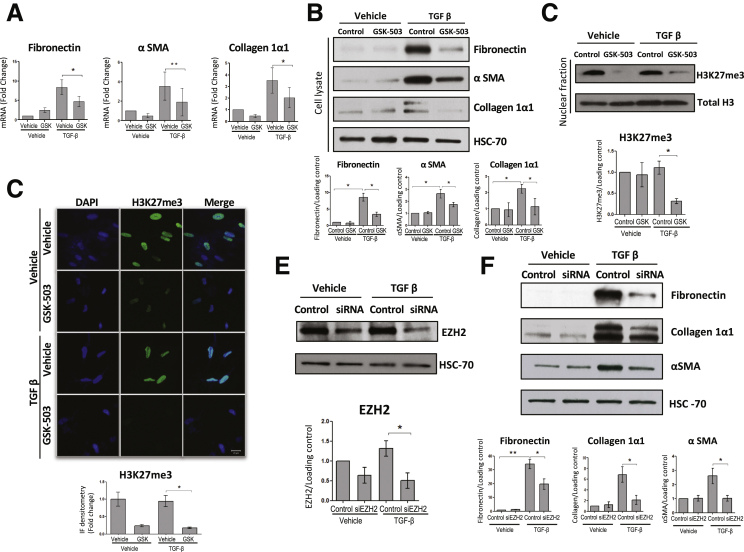
**EZH2 inhibition attenuates HSC activation.** Primary human HSCs were treated for 48h with GSK-503 and TGF-β (*A*) mRNA levels of fibronectin (*P* = .0407), αSMA (*P* = .0017) and collagen 1α1 (*P* = .0232) were significantly decreased after treatment with GSK-503 in those cells activated with TGF-β. (*B*) Western blots and quantification graphs of fibronectin (vehicle vs vehicle-TGF-β; *P* = .0252; vehicle-TGF-β vs GSK-503-TGF-β; *P* = .0206), αSMA (vehicle vs vehicle-TGF-β; *P* = .0466; vehicle-TGF-β vs GSK-503-TGF-β: *P* = .0452), and collagen 1α1 (vehicle vs vehicle-TGF-β; *P* = .0450; vehicle-TGF-β vs GSK-503-TGF-β; *P* = .0449) showing the same effect at the protein level. (*C*) H3K27me3 significantly decreased in HSCs treated with TGF-β and GSK-503 (*P* = .0486). (*D*) The effect of the drug in H3K27me3 was also demonstrated by immunofluorescence (*P* = .0268). (*E*) EZH2 expression was effectively reduced with EZH2 siRNA (*P* = .0494). (*F*) Western blots and quantification graphs showed a decrease in fibronectin (control-vehicle vs control-TGF-β: *P* = .0095; control-TGF-β vs siRNA-TGF-β; *P* = .0484), αSMA (*P* = .0455) and collagen 1α1 (*P* = .0171) with the EZH2 siRNA. Paired *t*-test. Data are expressed as means ± SEM. All *P* values represent at least 3 different experiments performed in triplicate. ∗*P* < .05; ∗∗*P* < .01. DAPI, 4′,6-diamidino-2-phenylindole.

We next sought to examine whether EZH2 inhibition, either with pharmacologic or genetic approaches, would modulate HSC activation in vitro. First, we used an epigenetic compound, GSK-503, which specifically targets the catalytic subunit of EZH2.^15^, ^16^ Inhibition of EZH2 in cells treated with GSK-503 and TGF-β led to a significant decrease in fibronectin, α-SMA, and collagen 1α1, both at mRNA and protein levels (Figure 3*A* and *B*). Parallel to this effect, we confirmed a significant down-regulation of H3K27me3 measured by Western blot and immunofluorescence (Figure 3*C* and *D*).

To further show the effect of EZH2 inhibition in HSCs, we knocked down the gene using siRNA. Primary human HSCs were incubated with SignalSilence EZH2 siRNA I (Cell Signaling Technology, Danvers, MA) for 48 hours, and then treated with TGF-β or vehicle for another 48 hours. EZH2 knockdown was confirmed at the protein level (Figure 3*E*) and, consistent with our previous results, down-regulation of EZH2 with the siRNA significantly attenuated the TGF-β–induced increase in fibronectin, α-SMA, and collagen 1α1 (Figure 3*F*). These results suggest that EZH2 inhibition contributes to attenuate TGF-β–dependent HSC activation and production of ECM proteins.

### EZH2 Overexpression Promotes Production of ECM Proteins In Vitro

**Figure 4:**
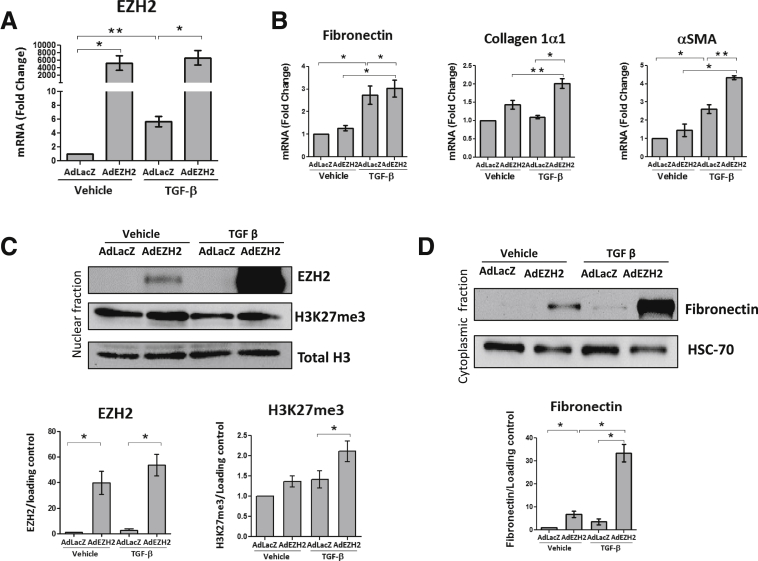
**EZH2 adenoviral overexpression promotes ECM protein production.** Primary human HSCs were infected with an adenovirus overexpressing EZH2 for 48 hours and then treated with TGF-β for another 48 hours. (*A*) Quantitative PCR confirmed the overexpression of EZH2 with the adenovirus (vehicle-AdLacZ vs vehicle-AdEZH2, *P* = .0294; vehicle-LacZ vs TGF-β-AdLacZ, *P* = .0072; and TGF-β-AdLacZ vs TGF-β-AdEZH2, *P* = .0406). (*B*) EZH2 overexpression significantly increased mRNA levels of fibronectin (vehicle-AdLacZ vs TGF-β-AdLacZ, *P* = .0494; vehicle-AdEZH2 vs TGF-β-AdEZH2, *P* = .0221; and TGF-β-AdLacZ vs TGF-β-AdEZH2, *P* = .0315), collagen 1α1 (vehicle-AdEZH2 vs TGF-β-AdEZH2, *P* = .0040; and TGF-β-AdLacZ vs TGF-β-AdEZH2, *P* = .0127), and α-SMA (vehicle-AdLacZ vs TGF-β-AdLacZ, *P* = .0188; vehicle-AdEZH2 vs TGF-β-AdEZH2, *P* = .0228; and TGF-β-AdLacZ vs TGF-β-AdEZH2, *P* = .0067). (*C*) EZH2 overexpression was confirmed at the protein level (vehicle-AdLacZ vs vehicle-AdEZH2, *P* = .0496; and TGF-β-AdLacZ vs TGF-β-AdEZH2, *P* = .0316) and paralleled the increase of H3K27me3 in cells treated with TGF-β (*P* = .0478) (*upper panel*: Western blot; *lower panel*: quantification graph). (*D*) Fibronectin protein expression increased as a result of the EZH2 adenovirus infection as shown by Western blot (*upper panel*) and the quantification graph (*lower panel*) (vehicle-AdLacZ vs vehicle-AdEZH2, *P* = .0493; vehicle-AdEZH2 vs TGF-β-AdEZH2, *P* = .0186; TGF-β-AdLacZ vs TGF-β-AdEZH2, *P* = .0264). Paired *t* test. Data are expressed as means ± SEM. All *P* values represent at least 3 different experiments performed in triplicate. ∗*P* < .05; ∗∗*P* < .01.

We next took an overexpression strategy to further investigate the effects of EZH2 in HSCs. We infected primary human HSCs with a plasmid encoding an overexpression mutant of EZH2 after 6 hours of serum starvation. As an experimental control, cells also were infected with an empty vector. After 48 hours, basal media was replaced and cells were treated with TGF-β for 48 hours. First, we confirmed the significant up-regulation of EZH2 at the mRNA level (Figure 4*A*), which led to a subsequent increase in fibronectin, α-SMA, and collagen 1α1 mRNA (Figure 4*B*). Finally, we confirmed that EZH2 adenoviral overexpression and the subsequent increase in H3K27me3 leads to a significant increase in fibronectin protein expression as well (Figure 4*C* and *D*).

### EZH2 Inhibition Attenuates Liver Fibrosis in Mice Treated With CCL_4_ and BDL

**Figure 5:**
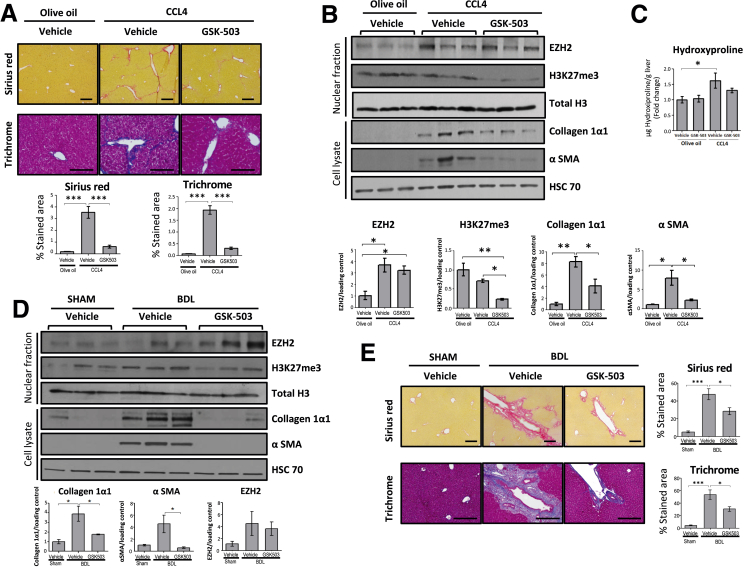
**EZH2 inhibition attenuates liver fibrosis in vivo.** C57/BL6 mice were treated with olive oil and either GSK-503 (n = 6) or vehicle (n = 6), and CCL_4_ and either GSK-503 (n = 6) or vehicle (n = 6) for 4 weeks. (*A*) Paraffin liver sections of mice treated with CCL_4_ or olive oil and GSK-503 or vehicle. CCL_4_-induced liver injury was attenuated by GSK-503 in Sirius red (5x) and Masson's Trichrome (20x) stain. (*B*) Western blots and quantification graphs from mice treated with CCL_4_ and GSK-503 showed a significant decrease in H3K27me3 and, subsequently, a significant decrease in collagen 1α1 and α-SMA. EZH2 increased significantly in mice treated with CCL_4_. (*C*) Collagen deposition measured by hydroxyproline assay was consistent with previous results. (*D*) C57/BL6 mice were subjected to BDL and treated with GSK-503 (n = 5) or vehicle (n = 6), or underwent sham intervention and received GSK-503 (n = 6) or vehicle (n = 6). Mice that were treated with GSK-503 showed a significant decrease in collagen 1α1 and α-SMA. EZH2 showed a nonsignificant increase in BDL group vs sham. (*E*) Paraffin-embedded liver sections were stained with Sirius red (5x) and Masson's Trichrome (10x). Data are expressed as means ± SEM. All statistical calculations were performed with analysis of variance and the Bonferroni comparison post-test. ∗∗∗*P* < .0001; ∗∗*P* < .005; ∗*P* < .05.

Next, we assessed the effect of EZH2 inhibition in vivo. Wild-type (WT) mice were treated with olive oil or CCL_4_ and GSK-503 or vehicle for 4 weeks. GSK-503 was administered intraperitoneally (IP) 3 times a week (1.5 mg/mouse). Administration of GSK-503 to mice attenuated CCL_4_-induced fibrosis as assessed by Masson's Trichrome and Sirius red stains from paraffin-fixed liver tissues (Figure 5*A*). At the protein level, we found a significant up-regulation of EZH2 in all mice treated with CCL_4_ compared with those who received olive oil (Figure 5*B*). However, the H3K27me3 mark was attenuated significantly in response to GSK-503 administration as assessed by Western blot (Figure 5*B*). Collagen 1α1 and α-SMA protein expression paralleled the H3K27me3 reduction (Figure 5*B*). Hydroxyproline measurement from tissue lysates also showed a reduction in collagen deposition in CCL_4_ plus GSK-503 administered mice as compared with mice receiving CCL_4_ alone (Figure 5*C*).

To corroborate the in vivo effect of EZH2 inhibition in an alternative model of liver fibrosis, we performed BDL in WT mice. Seven days after surgery, GSK-503 was administered IP every 48 hours (1.5 mg/mouse) and, finally, mice were killed 15 days after the intervention. Similar to the experiment with CCL_4_, mice subjected to BDL but treated with GSK-503 showed decreased collagen and α-SMA protein expression (Figure 5*D*). Sirius red and Masson's Trichrome stains also showed decreased ductal proliferation (Figure 5*E*). These in vivo results indicate that EZH2 inhibition attenuates murine liver fibrosis.

## Discussion

Delineation of redundant and distinct signaling downstream of canonical TGF-β and PDGF pathways is important for HSC biology and potential therapeutic interventions. In this study, we identify a new role of the HMT EZH2 in hepatic fibrosis through preferential induction by TGF-β as compared with PDGF-dependent pathways. We show that EZH2 inhibition attenuates HSC activation and fibrosis in vivo, and, conversely, EZH2 overexpression promotes ECM protein deposition. This is of particular interest given the number of EZH2 pharmacologic inhibitors that are under drug development and clinical evaluation, particularly in the neoplasia space.^17^, ^18^, ^19^, ^20^

TGF-β and PDGF are critically involved in HSC transdifferentiation. TGF-β is canonically the more notable profibrotic stimulus,^4^ whereas PDGF is more prominent for its mitogenic and motogenic effects on HSCs.^5^ The comparison analysis between HSCs treated either with TGF-β or PDGF showed a predominantly profibrotic role of TGF-β in HSC activation along with overlapping regulation of multiple genes with PDGF. Interestingly, the analysis also showed that this effect may be related to the fact that TGF-β stimulation also activates other growth factors involved in HSC activation, including PDGFA and B, CTGF, VEGFA, and fibroblast growth factor 2. Therefore, TGF-β plays a key role in HSC activation, and epigenetic modifications may contribute to this process. Some studies have explored the epigenetic regulation of TGF-β signaling in other biological contexts; it has been shown that DNA methylation,^21^, ^22^ microRNAs,^23^ and histone modifications^24^ regulate gene expression of key effectors of the pathway; however, evidence in liver fibrosis is scarcer.^25^ Therefore, the specific contribution of epigenetics on TGF-β-induced HSC activation was the focus of our current investigation.

There is a growing interest in epigenetic drug development for investigational and therapeutic purposes.^26^, ^27^ Given the emergence of the oncogene EZH2 from our RNA sequencing data set, we focused on the role of this HMT in TGF-β–induced HSC activation and liver fibrosis in vivo. Some prior studies also have addressed histone modifications and liver fibrosis including EZH2. For example, it has been reported that the histone methyltransferase inhibitor, 3-deazaneplanocin A, may halt the progression of liver fibrosis in mice treated with CCL_4_.^28^ However, 3-deazaneplanocin, although initially considered a selective EHZ2 inhibitor, showed poor specificity because it altered multiple histone methylation marks. Here, we show the specific role of EZH2 by means of a targeted knockdown with a siRNA. Mann et al^11^ also uncovered an interesting link between methyl CpG binding protein 2, miR132, and EZH2. They showed that EZH2 may contribute to peroxisome proliferator-activated receptor γ transcriptional silencing in a relay pathway along with methyl CpG binding protein 2 and microRNA132. It also has been reported that EZH2-mediated repression of Dkk-1 may promote activation of the Wnt/β-catenin pathway,^29^ which has been shown to contribute to HSC transdifferentiation.^30^ Our results consistently confirm the profibrotic role of EZH2 in vitro and in vivo, corroborating its potential as a target for antifibrotic therapy.

Several possible mechanisms may explain the profibrotic effect of EZH2. In addition to the previously mentioned role as a repressor of important negative regulators of liver fibrosis,^11^, ^29^ Xiao et al^12^ showed that inhibition of EZH2 also may reduce TGF-β–induced differentiation of human lung fibroblasts into myofibroblasts, preventing Smad2/3 nuclear translocation. On the other hand, it also has been shown that EZH2 mediates the development of renal fibrosis by down-regulating the expression of Smad7, a negative regulator of the TGF-β signaling pathway.^13^ Finally, in the liver, EZH2 also contributes to other processes implicated in fibrogenesis such as proliferation and epithelial-to-mesenchymal transition.^31^, ^32^ Therefore, it is conceivable that EZH2 promotes liver fibrosis by targeting different pathways, and in coordination with other epigenetic marks. Because of the predominant nuclear localization of EZH2, and the small percentage of HSCs in liver tissues, specific EZH2 staining of activated HSCs in human samples has been difficult, however, increased global expression of EZH2 has been shown in mice treated with CCL_4_ vs controls,^24^ and in patients with liver failure and liver cancer as compared with healthy controls.^33^, ^34^

EZH2 is well known for its role in H3K27me3, leading to chromatin compaction and gene silencing. However, chromatin-independent functions also have been shown. EZH2 may drive methylation of nonhistone proteins such as transcription factors involved in cell adhesion and migration,^35^ actin polymerization,^36^ or T-cell development,^37^ among others.^38^, ^39^ It is conceivable that these noncanonical roles of EZH2 also could contribute to the effects we observed.

Finally, multiple evidences have shown that EZH2 is highly expressed and plays a key role in several types of cancer.^9^, ^40^, ^41^, ^42^ It has been identified as one of the most deregulated epigenetic modulators in hepatocellular carcinoma (HCC),^10^, ^43^ promoting hepatocarcinogenesis through different mechanisms. Specifically, EZH2 contributes to liver cancer through modulation of different tumor-suppressor microRNAs,^44^, ^45^ long noncoding RNAs,^46^ or regulating the cell cycle,^47^ among other processes. It also has been shown that EZH2 gene expression levels in HCC specimens may have prognostic implications.^34^, ^47^, ^48^ Recently, some evidence has suggested that EZH2 inhibition contributes to HCC immune-mediated eradication by natural killer cells.^49^ Thus, EZH2 could represent a potential link between fibrosis and the ensuing HCC that frequently occurs in the fibrotic liver.

In summary, in this study, we present evidence that supports the role for the HMT EZH2 in promoting TGF-β– but not PDGF-dependent fibrogenic pathways. In addition, we show that EZH2 inhibition attenuates HSC activation in vitro and liver fibrosis in vivo. Consequently, EZH2 modulation may be a promising epigenetic target for liver fibrosis.

### Materials and Methods

#### Cell culture

Primary human HSCs (5300; ScienCell Research Laboratories, Carlsbad, CA) were grown in Dulbecco's modified Eagle medium (11965092; Life Technologies, Carlsbad, CA) containing 10% fetal bovine serum (FBS) (F4135; Sigma-Aldrich, St. Louis, MO) and 1% penicillin/streptomycin (15140122; Life Technologies). Cells were treated with GSK-503 (ApexBio, Houston, TX) (10 mmol/L) and/or TGF-β (240-B; R&D Systems, Minneapolis, MN) (10 ng/mL) and/or PDGF-BB (P3201; Sigma-Aldrich) (10 ng/mL) for 48 hours. All cell lines were maintained under standard tissue culture conditions (37°C, 5% CO_2_ incubator).

#### RNA sequencing

RNA sequencing and bioinformatics analyses were conducted in collaboration with the Mayo Medical Genomics Facility. The quality of the RNA was assessed by the Mayo Gene Expression Core using Agilent Bioanalyzers. RNA sequencing was performed as paired-end base reads on an Illumina HiSeq 2000 with 3 samples per lane, using the TruSeq SBS Sequencing Kit, version 3. Base calling was performed using Illumina’s (San Diego, CA) RTA version 1.12.4.2. Bioinformatics were performed with the assistance of the Mayo Division of Biostatistics and Informatics. Analysis of each sample (alignment statistics, in-depth quality-control metrics, and gene and exon expression levels) was performed using Mayo Clinic’s MAPRSeq (Rochester, MN) v1.2. Reads were mapped using Tophat (Baltimore, MD) version 2.0.6 against the hg19 reference genome, and gene counts were produced using high-throughput sequencing. Differential expression analyses between samples were computed using an edgeR version 3.3.8 algorithm. A whole-genome heat map was created with the Heatmapper web server as described elsewhere.^50^

#### ChIP sequencing

Liver tissue was collected from patients with alcoholic hepatitis undergoing early liver transplantation (n = 7), and from nondiseased livers (n = 5).^14^ ChIP sequencing was performed for H3K27me3 and data were processed using Mayo Clinic bioinformatics pipelines. Regions showing differential occupancy by individual histone marks were identified and assigned to promoters.

#### Quantitative reverse-transcription polymerase chain reaction

The RNeasy kit (Qiagen) was used to extract total RNA from cells (and mouse tissue) according to the manufacturer’s instructions. RNA was reverse-transcribed using the SuperScript III System (Invitrogen, Carlsbad, CA), and TaqMan-based real-time reverse-transcription polymerase chain reaction (PCR) was performed according to the manufacturer’s instructions (Applied Biosystems, Foster City, CA). Amplification of glyceraldehyde-3-phosphate dehydrogenase (or β-actin) was performed in the same reaction for the respective samples as an internal control. Each experiment was performed at least in triplicate. Real-time PCR conditions were as follows: 95ºC for 5 minutes, then 40 cycles of 95ºC for 15 seconds, 60ºC for 30 seconds, 72ºC for 30 seconds, and a final extension at 72ºC for 30 seconds. Mice and human primer sequences are listed in Table 2.

**Table 2:** Mice and Human Primer Sequences

h EZH2	Forward	5′-CCCTGACCTCTGTCTTACTTGTGGA-3′
	Reverse	3′-ACGTCAGATGGTGCCAGAAATA-3′
h Fibronectin	Forward	5’-GATAAATCAACAGTGGGAGC-3’
	Reverse	5’-CCCAGATCATGGAGTCTTTA-3’
h α-SMA	Forward	5’-GACAGCTACGTGGGTGACGAA-3’
	Reverse	5’-TTTTCCATGTCGTCCCAGTTG-3’
h Collagen 1α1	Forward	5’-CCCGGGTTTCAGAGACAACTTC-3’
	Reverse	5’-TCCACATGCTTTATTCCAGACATC-3’
h GAPDH	Forward	5’-CCAGGGCTGCTTTTAACTCT-3’
	Reverse	5’-GGACTCCACGACGTACTCA-3’
m β-actin	Forward	5’-AGAGGGAAATCGTGCGTGAC-3’
	Reverse	5’-CAATAGTGATGACCTGGCCGT-3’
m Collagen 1α1	Forward	5’-GAGCGGAGAGTACTGGATCG-3’
	Reverse	5’-GCTTCTTTTCCTTGGGGTTC-3’
m EZH2	Forward	5’-TCCCGTTAAAGACCCTGAATG-3’
	Reverse	5’-TGAAAGTGCCATCCTGATCC-3’
m Fibronectin	Forward	5’-GTGGCTGCCTTCAACTTCTC-3’
	Reverse	5’-GTGGGTTGCAAACCTTCAAT-3’
m α-SMA	Forward	5’-AAACAGGAATACGACGAAG-3’
	Reverse	5’-CAGGAATGATTTGGAAAGGA-3’
m EZH2	Forward	5’-TCCCGTTAAAGACCCTGAATG-3’
	Reverse	5’-TGAAAGTGCCATCCTGATCC-3’

GAPDH, glyceraldehyde-3-phosphate dehydrogenase; h, human; m, mouse.

#### Western blot analysis

HSCs or liver tissues were lysed and prepared for Western blot analysis as described previously.^51^ Immunoblot analysis was performed according to the protocol recommended for individual antibodies as listed in Table 3. Immunoreactive bands were visualized using horseradish-peroxidase–conjugated secondary antibody and an enhanced chemiluminescent system (sc-2048, Immuno Cruz; Santa Cruz Biotechnology, Inc, Dallas, TX; or WBLUR0100, Luminata Crescendo, Millipore Sigma, Burlington, MA). For some experiments, the protein nuclear fraction was isolated and lysates then were sonicated for 15 seconds (amplitude, 5%).

**Table 3:** Antibodies Used in Western Blot Analysis

Primary antibody	Company and product number
EZH2	Cell Signaling 5246
H3K27me3	Abcam Ab6147
GAPDH	Invitrogen AM4300
Fibronectin	Santa Cruz sc-9068
HSC 70	Santa Cruz sc-7298
Collagen 1	Southern Biotech (Birmingham, AL) 1310-01
α-SMA	Abcam ab5694
Histone 3	Abcam ab1791

GAPDH, glyceraldehyde-3-phosphate dehydrogenase.

#### Cell immunofluorescence

HSCs were plated at 80% confluence on 8-well chamber slides (177402; Lab-Tek, Sigma-Aldrich) precoated with collagen (C4243; Sigma-Aldrich). After 24 hours of culture, cells were washed with 1× phosphate-buffered saline and incubated with GSK-503 (ApexBio) (10 mmol/L) and/or TGF-β (240-B; R&D Systems) (10 ng/mL) for 48 hours. Cells then were fixed in 4% paraformaldehyde and nonspecific sites were blocked with 10% FBS. Cells then were incubated overnight at 4ºC in primary antibody to detect H3K27me3 (1:200) (ab6147; Abcam, Cambridge, United Kingdom) diluted in 1% bovine serum albumin. After washing, cells were incubated in fluorochrome-coupled secondary antibody diluted in 1× phosphate-buffered saline for 1 hour at room temperature. All secondary antibodies were provided by Life Technologies and used at 1:200.

#### RNA interference knockdown

After overnight starvation, human HSCs were transfected with 100 nmol/L of siRNA (SignalSilence Ezh2 siRNA I 6509; Cell Signaling Technology) using Oligofectamine (12252011; Invitrogen) according to the manufacturer’s instructions. Forty-eight hours later, media was replaced and TGF-β (240-B; R&D Systems) (10 ng/mL) was added for 48 hours.

#### Adenoviral transfection

Epitope-tagged (6XHis-Xpress) EZH2α was generated as recombinant adenovirus by the Gene Transfer Vector Core at the University of Iowa. Empty vector (pacAD5 CMV) was used as the experimental control as described elsewhere.^52^ Primary human HSCs were infected with the adenovirus (8^10^ pfu/mL) after a 6-hour starvation. Forty-eight hours later, 1% FBS Dulbecco's modified Eagle medium was replaced and cells were treated with TGF-β (240-B; R&D Systems) (10 ng/mL) for another 48 hours.

### Liver Fibrosis In Vivo Models

#### CCL_4_ and GSK-503 treatment

WT C57/BL6 mice (age, 8 wk) were purchased from Envigo (Huntingdon, United Kingdom). We chose female mice because to their higher susceptibility to liver injury.^53^ CCL_4_ was injected IP twice a week at 4 μL/g/body (CCL_4_/olive oil, 1:3) for 4 weeks. GSK-503 also was administered IP 3 times a week (1.5 mg/mouse) alternating with CCL_4_ on different days. Twenty-four hours after the ﬁnal injection, mice were killed and livers were harvested. Fibrosis was analyzed by Sirius Red and Masson's Trichrome stains (parafﬁn-embedded tissues), hydroxyproline analysis, quantitative PCR, immunoblotting, and immunoﬂuorescence. All animal experiments followed protocols approved by the Mayo Clinic Institutional Animal Care and Use Committee.

#### BDL model

BDL was performed as previously described^54^ in WT 8-week-old female mice. Seven days after the surgery, the animals were injected IP with GSK-503 (1.5 mg/mouse) every 48 hours and killed 15 days later.

#### Hydroxyproline assay

Hepatic hydroxyproline levels were quantified using a colorimetric assay. Frozen liver tissues (50–100 mg) were hydrolyzed in 6 N hydrochloric acid at 100°C for 18 hours. Samples then were dried using a speed vacuum overnight. The precipitates were suspended in distillated water and transferred to 0.22-μm–filter centrifuge tubes. In duplicates, 5 μL of the filtered samples were incubated with 50 μL of chloramine-T and distillated water for 20 minutes. Then, 50 μL of Elrich–perchloric acid was added and incubated for 15 minutes at 65°C and 20 minutes at room temperature. The absorbance was read at 561 nm using a spectrophotometer, and the results were normalized by the weight of each sample.

### Statistical Analysis

Results are expressed as the means of 3 or more independent experiments. Analysis of variance with Bonferroni post-test, nonparametric 2-tailed *t* test (Mann–Whitney), or paired *t* test for cell culture experiments were used to assess the statistical significance between groups as appropriate with GraphPad Prism 5 software (GraphPad Software, Inc, La Jolla, CA). A *P* value less than .05 was considered statistically significant.

All authors had access to the study data and reviewed and approved the final manuscript.
